# Beyond lactation: Central oxytocin in maternal mental health and disease

**DOI:** 10.1016/j.neubiorev.2026.106697

**Published:** 2026-07

**Authors:** Eduard Maier, William Lan, Stephanie Schimmer, Shimpei Ishiyama, Valery Grinevich

**Affiliations:** a Department of Neuropeptide Research in Psychiatry, Central Institute of Mental Health, Medical Faculty Mannheim, University of Heidelberg, Germany; b German Center for Mental Health (DZPG), Mannheim, Heidelberg, Ulm, Germany; c International Joint Laboratory for Translational Research on Neuromodulation, Shenzhen Institutes of Advanced Technology, Chinese Academy of Sciences, Shenzhen, China

**Keywords:** Oxytocin bursting, Maternal brain, Lactation, Breastfeeding, Stress regulation, Postpartum depression

## Abstract

This review summarizes the literature on the bursting of oxytocin (OT) neurons during lactation, their downstream mechanisms of action in the brain, and potential implications for this neuropeptide on maternal mental health. We describe how OT bursts likely affect various brain areas, each containing unique compositions of OT fibers, OT receptors (OTRs), and OTR-expressing cell types. Burst firing of OT neurons leads to non-linear OT secretion and, in turn, downstream molecular pathways following OTR activation also appear non-linear. Such a nested, switch-like relationship suggests that OT bursts mediate profound changes in maternal brain function. In line with this hypothesis, most human studies report that breastfeeding and the associated OT response to suckling have protective effects against postpartum depression (PPD). Conversely, PPD can negatively affect breastfeeding success. We scrutinize mechanistic pathways that may contribute to these interactions and propose direct pathways for interactions between the OT and the stress system, which may play a role in PPD pathophysiology. We also speculate about indirect pathways involving sensorimotor areas, which are optimized by the OT system to prevent frustration and subsequent negative stress effects on caregiving. Finally, we analyze publication trends and study types, identifying a surge in breastfeeding research in recent years that is not reflected in the animal literature. Most studies examine OT, breastfeeding, and related pathologies in isolation, which highlights the need for more holistic approaches in both human and animal research.

## Highlights

• Oxytocin (OT) neuron bursts are a hallmark of the lactation period.
• Central burst evoked OT release is distinct form non-burst release.
• OT bursts may affect multiple brain regions with distinct receptors and cell types.
• OT bursts during breastfeeding may help prevent postpartum depression (PPD)
• New animal studies are essential to dissect OT-related mechanisms of PPD

## 1 Introduction

Before the neurohormone oxytocin (OT) was isolated and synthesized by Vincent du Vigneaud in the 1950s (Du Vigneaud, 1956), its effects on uterine contractions and milk ejection had already been demonstrated through the application of hypophyseal extracts in the early 20th century (Dale, 1906, Ott and Scott, 1910). It is now established that OT is secreted from the posterior pituitary into the bloodstream in response to suckling. Circulating OT subsequently binds to OT receptors (OTRs) expressed on myoepithelial cells surrounding the mammary alveoli and ducts, triggering their contraction and thereby facilitating milk ejection (Liu et al., 2020).

For two decades following the work of Vincent du Vigneaud, milk ejection and uterine contraction were widely regarded as the principal functions of oxytocin in maternal peripheral physiology. This view began to change in the late 1970s, when Pedersen and Prang (Pedersen and Prange, 1979) demonstrated that central administration of oxytocin could induce the onset of maternal behavior in rats, providing some of the first compelling evidence for a profound role of oxytocin within the brain.

**Fig. 1:**
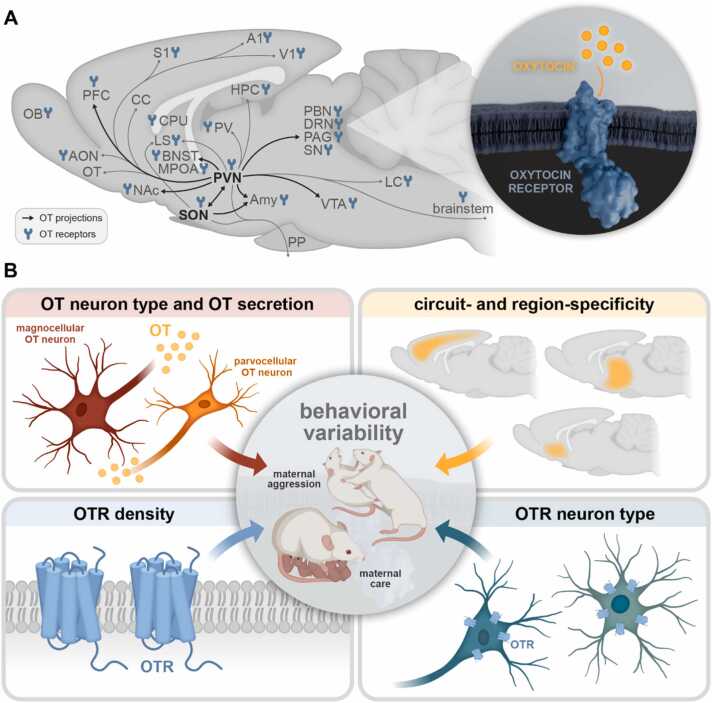
Operating principles of OT signaling in the brain. A. Large-scale organization of the OT system, exemplified in the rat brain. OT fibers and OTRs are distributed across various brain areas. Adapted from (Grinevich and Neumann, 2021). B. Each brain area contains a “Mini-OT system” with its own OT machinery, comprising a unique combination of OT secretion strength, OTR density, and OTR-expressing cell types, thereby explaining the variety of physiological and behavioral changes elicited by this neurohormone/neuropeptide.

While the central effects of OT on maternal behavior were evident across various brain areas using pharmacological approaches and radioligand binding assays (reviewed in (Pedersen, 1997)), it remained unclear how OT reaches its target brain areas. This changed with the development of viral vectors enabling manipulation of OT neural activity and brain-wide axonal projection analysis of OT neurons, which revealed OT axons in a variety of forebrain, midbrain/interbrain, and hindbrain areas (Knobloch et al., 2012, Grinevich and Neumann, 2021) (Fig. 1A). Similarly, experiments using transgenic animal lines (Newmaster et al., 2020), in situ hybridization (Li et al., 2025, Ryu et al., 2026), immunohistochemistry (Mitre et al., 2016), and radioligand binding assays (Insel and Shapiro, 1992, Young et al., 1996, Witt et al., 1991) revealed a widespread OTR expression pattern. While the OT system may appear similar across brain areas at first glance (Fig. 1A), its net function varies greatly depending on OT secretion, OTR density, and OTR-expressing cell types within a given area (Fig. 1B). This has been demonstrated in recent studies using modern genetic, viral, and molecular tools. For example, Knobloch et al. (2012) showed that axons from OT neurons project differently across brain areas. Newmaster et al. (2020) and Mitre et al. (2016) demonstrated that OTR expression varies greatly across regions, while Schimmer et al. showed that OT promotes prosocial behavior in a circuit-dependent manner (Schimmer et al., 2026).

This review aims to illuminate how such area-dependent “Mini-OT systems” operate in healthy mothers and how this may contribute to a better understanding of pathological conditions in which mother-child interactions are perturbed, such as postpartum depression (PPD).

**Fig. 2:**
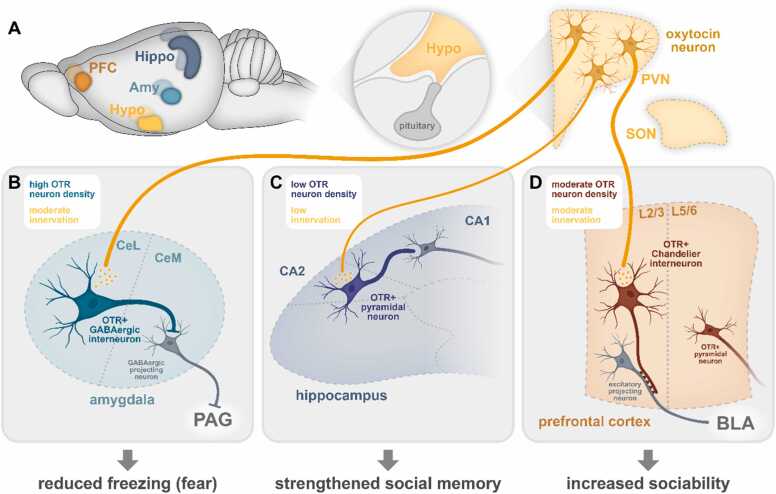
Behaviorally relevant Mini-OT system example circuits. A. Overview of the relevant brain areas (left) and the location of PVN neuron cell bodies within the PVN (middle and right). B. OT projections to the central amygdala (CeL) are moderate but stronger than those to other brain areas (see example in C). These projections terminate on GABAergic OTR-expressing interneurons, which in turn innervate GABAergic periaqueductal gray (PAG)-projecting neurons in the medial amygdala (CeM). This circuit has been shown to control fear-related behaviors, such as freezing. C. OT projections to the hippocampus and OTR-expressing cells within this region are relatively sparse. Nevertheless, CA2 OTR-expressing glutamatergic (pyramidal) neurons projecting to CA1 allow memory formation. D. The OT projection density to the prefrontal cortex is moderate and comparable to the CeL projections shown in B. The OTR cell density is moderate and largely restricted to the superficial layers (L2/3). OTR-expressing chandelier interneurons target the axon initial segment of glutamatergic basolateral amygdala (BLA)-projecting neurons. This circuit controls sociability. Note that these circuits are based on several publications (Knobloch et al., 2012, Newmaster et al., 2020, Schimmer et al., 2026, Althammer et al., 2022, Lin et al., 2018, Tsai et al., 2022), which used mice and rats as model organisms.

The OT system is unique among peptidergic circuits in the brain. Its principal distinction lies in the fact that OT-producing neurons are exclusively localized to a single brain region: the hypothalamus. In contrast, most other neuropeptides are broadly distributed throughout the mammalian central nervous system (Grinevich and Dobolyi, 2021). Despite their relatively small number, OT neurons form extensive extrahypothalamic projections, releasing OT at distant terminals and acting on relatively small populations of target cells, predominantly GABAergic neurons, with some notable exceptions. Through this mode of action, OT modulates local circuit activity, biasing network dynamics toward specific behavioral outputs. As mentioned above, we refer to these functionally distinct modules as Mini-OT systems. Each of these subsystems supports distinct behavioral functions, including social analgesia, social memory, and anxiolysis, collectively contributing to prosocial behavior and ultimately to species propagation. Fig. 2 illustrates this concept by presenting three distinct OT-dependent circuits, highlighting how OT exerts region-specific effects across the brain and how each Mini-OT system (OT subsystem) maps onto distinct behavioral outcomes. The diversity of Mini-OT systems is illustrated by distinct OT innervation patterns, OTR expression profiles, and the involvement of different OTR-expressing cell types (Fig. 2). As illustrated, OT projection densities, OTR cell densities, OTR-expressing cell types, and the resulting circuits are highly dependent on the brain region studied and give rise to distinct behavioral effects.

## 2 Physiology of the OT system during lactation

### 2.1 OT plasma levels across pregnancy and lactation

**Fig. 3:**
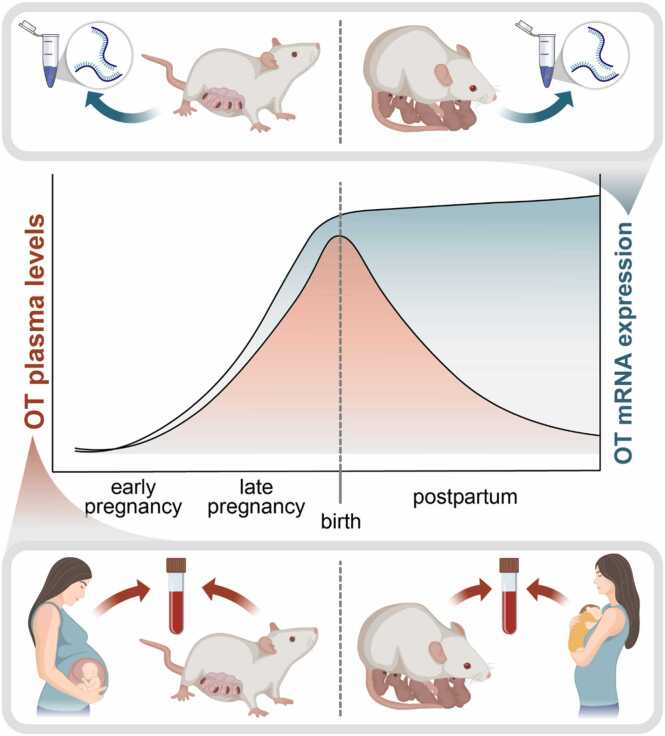
Relationship between peripheral baseline OT plasma levels and OT mRNA expression across pregnancy and the postpartum/lactation period. In animals and humans, baseline OT plasma levels rise until birth and then drop again (black line) (Prevost et al., 2014, Evans and Olley, 1988, Johnston and Amico, 1986). To our knowledge, OT transcription across gestation and lactation (blue line) has only been investigated in rats (Zingg and Lefebvre, 1988). Peripheral OT plasma levels do not entirely reflect OT synthesis. Note that this graph reflects qualitative rather than quantitative changes, and the curves are not drawn to scale. Additionally, the steepness of the rise and fall is difficult to estimate due to a lack of temporally resolved longitudinal studies. Curves were estimated based on refs (Vasicka et al., 1978, Dawood et al., 1979, Otsuki et al., 1983, Prevost et al., 2014, Zingg and Lefebvre, 1988, Evans and Olley, 1988, Johnston and Amico, 1986). Note that it is currently unclear whether or to which extent human and rodent OT levels differ, since OT plasma measurements vary greatly depending on the analytics used in the respective laboratories. As OT plasma levels rise dramatically upon suckling (Fuchs et al., 1991, Drewett et al., 1982, McNeilly et al., 1983, Jonas et al., 2009) (not shown here), this response should be considered in addition to the depicted basal plasma levels when estimating the effects of OT on the maternal brain.

With the onset of motherhood, both physiology and behavior change dramatically, which is also reflected in the OT system. OT plasma levels rise during pregnancy (Vasicka et al., 1978, Dawood et al., 1979, Otsuki et al., 1983) but drop again in the postpartum period (Prevost et al., 2014) (Fig. 3; for comprehensive reviews on OT plasma levels across pregnancy and the postpartum period, see (Uvnäs-Moberg et al., 2019, Uvnas Moberg et al., 2020)). These changes in OT levels are likely driven by sex hormone influences (reviewed in (Quintana et al., 2024)). Prevost et al. (2014) describe that OT levels show substantial variability (up to almost 100-fold differences across individuals) and that not all women exhibit the rise-fall pattern from before to after parturition. To our knowledge, it remains unknown whether these differences reflect underlying variation in maternal biology across individuals. Importantly, Prevost et al., 2014 did not ensure that sampling occurred during breastfeeding sessions. This is relevant, as studies that investigated OT plasma levels in a temporally resolved manner have shown peaks in secretion during pregnancy and parturition (Fuchs et al., 1991), as well as during breastfeeding (Drewett et al., 1982, McNeilly et al., 1983, Jonas et al., 2009). Therefore, basal plasma OT levels (i.e., samples obtained outside breastfeeding sessions) do not capture the magnitude or temporal structure of the transient OT surges triggered by breastfeeding. In this review, we primarily focus on these phasic, breastfeeding-associated increases. Nevertheless, longer-term changes in basal OT concentrations across pregnancy and the postpartum period may reflect slower regulatory processes with potentially distinct biological roles. Indeed, a few studies have compared basal OT levels with those obtained during breastfeeding by examining their respective associations with depressive symptoms; these studies are summarized in Section 3.2 and Table 1.

### 2.2 Discrepancy between OT synthesis in the hypothalamus and basal OT concentrations in blood

In rats, OT mRNA levels in the hypothalamus exhibit similar, but not identical, temporal dynamics (comparable human data are unavailable). OT mRNA levels rise throughout pregnancy but, instead of dropping after parturition, remain approximately threefold higher compared to those of virgin animals (Zingg and Lefebvre, 1988). However, this sustained increase is not reflected in basal rat OT plasma levels, as noted earlier (i.e., a decrease in baseline OT plasma levels after parturition; see (Prevost et al., 2014)). In fact, lactating rats appear to exhibit lower basal plasma OT levels compared to virgin animals (Evans and Olley, 1988), and in humans, basal plasma OT levels do not differ between breastfeeding and non-breastfeeding mothers (Johnston and Amico, 1986) (Fig. 3). This discrepancy between hypothalamic mRNA expression and plasma OT concentration likely reflects regulation at different temporal scales: increased mRNA levels during lactation ensure long-term OT availability, while short-term OT secretion is tightly restricted under basal conditions and strongly facilitated following infant suckling-induced OT bursting. Since OT peptide storage in vesicles is abundant (Lincoln and Paisley, 1982), increased mRNA expression may not have significant effects on short-term release quantities.

This highlights the need for caution when interpreting plasma OT levels, which may not reliably reflect actual OT availability or the magnitude of breastfeeding-induced OT release. Instead, transient OT responses to infant cues may represent a more relevant parameter for understanding oxytocin-dependent changes in maternal physiology and behavior.

### 2.3 OT neuronal activity during lactation

**Fig. 4:**
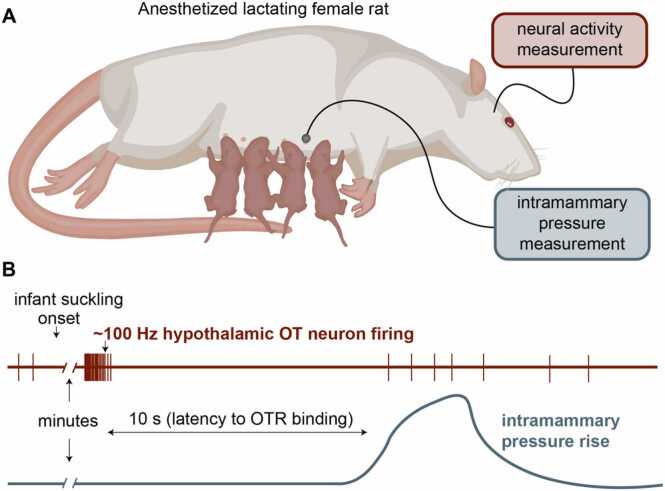
Neuronal firing patterns of a putative oxytocin neuron and simultaneously recorded intramammary pressure. A. Experimental setup. To our knowledge, combined recordings of OT neuronal activity and intramammary pressure have only been performed in anesthetized rats, as depicted here. **B.** Top: High-frequency bursts of action potentials are brief (1–3 s) and are followed by a ∼10 s period during which firing is absent. Bottom: A rise of intramammary pressure is detected approximately 10 s following high-frequency bursts. This suggests that approximately 10 s elapse between OT release from the pituitary and OTR binding in the mammary gland, which is dependent on the circulation properties of the studied individual/species. Adapted from Lincoln and Wakerley (1975), with permission.

The response of OT neurons to suckling was initially investigated in a series of studies by Wakerley and Lincoln in the 1970s (Wakerley and Lincoln, 1973, Lincoln and Wakerley, 1975) (Fig. 4), who found that, following suckling onset, putative OT neuronal activity increases from a low baseline (∼5 Hz) to firing rates of ∼100 Hz. These bursts are synchronized across OT neurons (Yaguchi et al., 2024), last only a few seconds, and recur at intervals of several minutes, suggesting the presence of a refractory period following high-frequency bursting. Simultaneous intramammary pressure recordings showed that the bursts are followed by an increase in pressure approximately 10 s thereafter.

In more recent studies, OT neuronal populations were recorded chronically. The authors reported that OT burst amplitudes and durations increase with the progression of lactation (Yaguchi et al., 2024, Yukinaga et al., 2022). At the same time, OT bursts occur in a more clustered manner (Yaguchi et al., 2023). These changes are thought to reflect the demands of momentary milk ejection: in later stages of lactation, mothers spend less time in the nest, while the caloric demands of older pups are higher. Milk is thus thought to be ejected more efficiently (higher and broader OT bursts) and more frequently (shorter inter-peak intervals) (Yukinaga and Miyamichi, 2025). Notably, while these studies primarily focus on OT bursting and its effects on milk ejection, they do not investigate the sites and effects of OT release in the brain.

### 2.4 Central OT release

#### 2.4.1 In-vivo recordings to identify brain area release sites

**Fig. 5:**
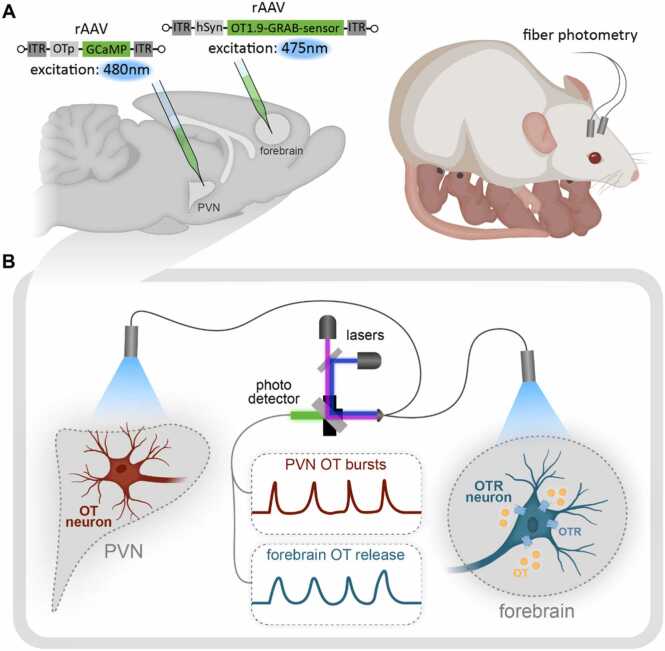
Future experiments to elucidate OT secretion in the brain enabled by novel fluorescence-based OT sensors (Qian et al., 2023, Ino et al., 2022). A. Sagittal rodent brain section illustrating a possible viral injection strategy. B. OT bursts can be recorded, as demonstrated in (Yukinaga et al., 2022, Yaguchi et al., 2023), and OT sensor signals from brain areas of interest (e.g., forebrain regions) can be aligned to these bursts to determine release kinetics and magnitude.

A number of studies using microdialysis have reported increases in OT levels following pup suckling in or adjacent to OT-containing hypothalamic nuclei (PVN (Bealer and Crowley, 1998, Neumann et al., 1993) and SON (Neumann et al., 1993, Neumann et al., 1993, Neumann et al., 1994, Moos et al., 1989)). Furthermore, OT release following suckling has been reported in extra-hypothalamic areas, including the septum and hippocampus (Neumann and Landgraf, 1989), as well as the medial preoptic area and the bed nucleus of the stria terminalis (Kendrick et al., 1992). While these studies suggest that OT is widely distributed throughout the brain following suckling, to the best of our knowledge, empirical studies investigating suckling-induced OT release in many other brain areas (Fig. 1A) and their area-specific functional roles are still lacking. Furthermore, despite providing valuable insights, the microdialysis method has relatively coarse temporal resolution (on the order of minutes, up to 30 min (Neumann et al., 1994)), resulting in inconclusive release kinetics. This will likely change with recent developments in fluorescent transmitter sensors, including novel OT sensors, which can be imaged in freely behaving animals (Qian et al., 2023, Ino et al., 2022) and provide sub-second resolution (Qian et al., 2023). As fiber photometry permits multisite recordings, simultaneous measurements of OT bursts (as demonstrated by calcium imaging (Yaguchi et al., 2024, Yukinaga et al., 2022, Yaguchi et al., 2023)) and OT sensor activity could, in principle, be obtained across multiple distant brain areas. It can therefore be expected that such experiments will be performed in the near future (Fig. 5).

Another open question is whether central and peripheral OT release originate from the same OT neurons. Traditionally, this was thought to be largely the case, as experiments in rats showed that pituitary-projecting magnocellular neurons also project to various forebrain areas (Zhang et al., 2021). However, recent high-resolution anatomical tracing experiments in mice (Li et al., 2024) indicate that pituitary- projecting OT neurons are distinct from forebrain-projecting OT neurons. It is unknown whether these discrepancies arise from methodological differences or species-specific differences in the composition of the OT system in rats versus mice.

As discussed above, OT bursts intensify across the lactation period. It remains unclear whether the increased bursting activity (Yaguchi et al., 2024) also translates into heightened OT secretion in target brain areas. This issue will likely be addressed by future experiments using the novel OT sensors described above (Fig. 5).

#### 2.4.2 Ex-vivo recordings reveal OT release facilitation during bursting

**Fig. 6:**
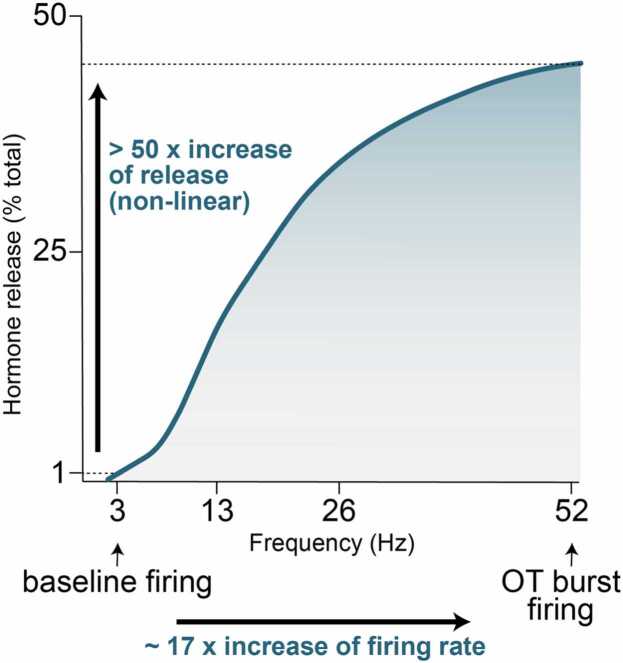
Non-linear increase of OT release after burst firing. Adapted from Bicknell (1988) with permission). Similar curves were observed in Bondy et al. (1987). Peptide release was measured by radioimmunoassay. Note that such non-linear OT release leads to disproportionately higher OT release following OT bursts in lactating animals.

In vivo studies in lactating rodents indicate that OT bursts are relatively rare events, with an inter-burst interval of approximately 5 min (Wakerley and Lincoln, 1973, Yukinaga et al., 2022). Thus, one may ask whether a short (1–3 s) acceleration in firing rate (from a baseline of 1–3 Hz to 75–100 Hz during bursting) makes a significant contribution to the overall amount of OT secretion. The answer to this question is provided by ex vivo recordings (Bicknell, 1988, Bondy et al., 1987), which demonstrate a non-linear relationship between OT firing rate and OT secretion. These data suggest an approximately 50-fold increase in OT release during bursts compared to baseline firing, despite only an approximately 17-fold increase in firing rate (Fig. 6; note that the firing rate increase was conservatively estimated from 3 Hz to 52 Hz). This means that a 1–3 s burst releases as much OT as the same neuron would release during 50–150 s of baseline firing. This corresponds to 16–50% of the baseline-only OT secretion calculated over the 5-minute interval separating two bursts. In other words, if similar mechanisms apply in humans, mothers who nurse and experience milk ejections driven by OT bursting may, on average, release up to 50% more OT during breastfeeding than mothers who do not nurse but have equivalent durations of tactile contact with their infants. These transient release events (e.g., 50-fold increases relative to baseline) may exert strong effects on downstream OTR functions and could therefore be particularly relevant for maternal health and disease.

In summary, OT is released not only into the circulation but also in various brain areas following suckling onset, and OT release in these areas is likely higher during episodes that include OT bursts. Novel imaging techniques will enable more accurate characterizations of OT bursting → OT release kinetics, as they are sufficiently sensitive to detect OT release in brain areas not previously investigated.

### 2.5 OTR expression and OTR function

OT mainly acts via OTRs, which are G-protein-coupled receptors first cloned in 1992 (Kimura et al., 1992). OTR activation engages various intracellular signaling pathways that lead to cell depolarization and activation of complex, cell type-dependent cascades that ultimately converge on transcription factor-mediated gene expression changes (reviewed in (Jurek and Neumann, 2018)).

Across pregnancy, OTR expression in the rat uterus has been shown to increase 25-fold and drop sharply after parturition (Larcher et al., 1995). Similar dynamics, although much less pronounced, have been observed centrally (Meddle et al., 2007, Insel, 1990, Insel, 1986, Naik and de Jong, 2017), and in some brain areas, higher OTR expression levels correlate with the degree of maternal behavior (Francis et al., 2000, Champagne et al., 2001). The most comprehensive brain-wide analysis comparing OTR expression between virgin and lactating mice was conducted by Mitre and colleagues (Mitre et al., 2016). The authors found that OTR-expressing cell numbers and densities (identified by antibody staining) varied across brain areas. Interestingly, when focusing on cortical areas, only a few displayed large differences between virgin and lactating animals. Nevertheless, despite this lack of large differences in OTR-expressing cell numbers between virgins and mothers, a series of studies in the auditory cortex (Carcea et al., 2021, Marlin et al., 2015, Schiavo et al., 2020) show that OT and OTRs play essential roles in driving appropriate maternal behavior.

**Fig. 7:**
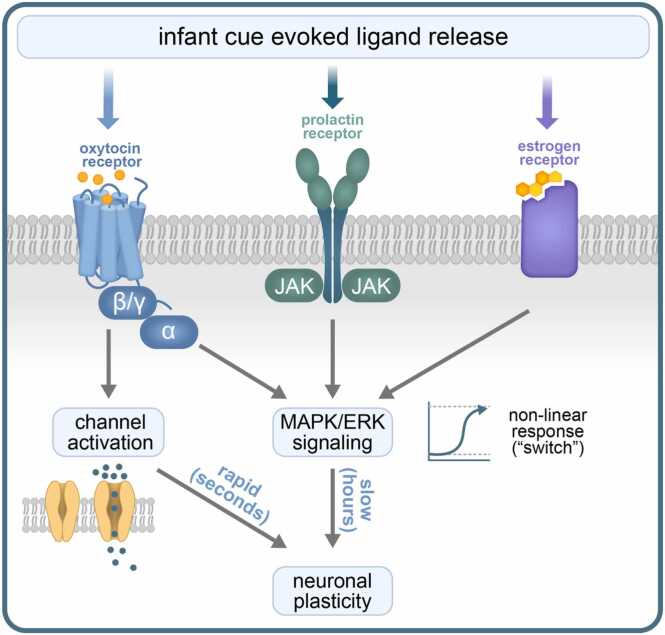
Possible interactions between neuroendocrine signaling pathways and sensory input. Individual studies have shown activation of MAPK/ERK following oxytocin, prolactin, and estrogen receptor activation (see text and (Zhong et al., 2003, Filardo et al., 2000, Singer et al., 1999, Blume et al., 2009, Kavarthapu et al., 2021, Aksamitiene et al., 2011)). However, to our knowledge, studies investigating these pathways simultaneously, particularly with respect to sensory-evoked neural plasticity, are lacking. Note that OTR signaling is initiated following local OT release and subsequent diffusion from axonal terminals (Chini et al., 2017) (OTR effects can therefore take minutes following activation of OT terminals (Knobloch et al., 2012)). OTR binding leads to the activation of different types of channels via G-protein mediated signaling, which in turn induces relatively rapid (within seconds to tens of seconds) changes in neuronal firing rates (Jurek and Neumann, 2018, Liu et al., 2022). This contrasts with slower transcriptional effects mediated by MAPK/ERK signaling (within hours) (Devost et al., 2022, Molnár et al., 1999). The MAPK / ERK signaling pathway shows a switch-like non-linear response to upstream signals (see main text). Additionally, channel effects can be mediated via G-alpha or G-beta/gamma subunits, while transcriptional effects typically require the G-alpha subunit (Gimpl and Fahrenholz, 2001). As OT release exhibits a non-linear relationship with OT firing (see previous figure), the combined release and intracellular properties may serve as filters favoring OT bursts over regular OT firing patterns.

Given the relatively small differences in cortical OTR expression between virgins and mothers, it is likely that additional mechanisms are modified across the maternal period to account for OT-dependent effects, independently of changes in OTR-expressing cell numbers in the cortex. We propose the following possibilities, which we hope will be further scrutinized in future experiments:

1. OT release at target sites may be increased in lactating animals in a non-linear manner due to burst firing (see previous sections).

2. OTR signaling may amplify downstream responses, similar to the non-linear relationship between OT release and OT burst firing (see previous sections). For example, in myometrial cells, OT can activate the Mitogen-Activated Protein Kinase/Extracellular signal-regulated Kinase (MAPK/ERK) pathway (Zhong et al., 2003) via OTR signaling and second messengers (see Jurek and Neumann, 2018 for extensive review (Jurek and Neumann, 2018) and Fig. 7). The MAPK/ERK pathway displays steep dose-response curves with a Hill coefficient between 4 and 5, suggesting that small changes in upstream input can produce large downstream effects (Huang et al., 1996). In line with this, OT shows a non-linear relationship with MAPK/ERK-dependent effector proteins (Devost et al., 2022). If this logic holds true in central OTR- expressing cells, OT release and OTR signaling in combination may ‘boost’ nursing-induced OT burst effects.

3. Intracellular pathways of different factors may converge with OTR signaling, thereby boosting their effects as well. For example, prolactin and estrogen signaling pathways can also lead to activation of the MAPK/ERK pathway (Fig. 7) (Zhong et al., 2003, Filardo et al., 2000, Singer et al., 1999, Blume et al., 2009, Wouters et al., 2014, Kavarthapu et al., 2021, Aksamitiene et al., 2011). Note that although circulating estrogen concentrations drop sharply after parturition (Dukic et al., 2024), some evidence points to an involvement of the estrogen *receptor* (potentially independent of estrogen itself (Gangolli et al., 1997)) in mediating maternal behavior during the lactation period (Champagne et al., 2003, Lonstein et al., 2000). Also note that pregnancy hormones, including estrogen and progesterone, correlate with postpartum brain volume remodeling (Niu et al., 2025, Servin-Barthet et al., 2025), which in turn is associated with reduced mother-infant attachment (Servin-Barthet et al., 2025, Hoekzema et al., 2017). Whether or how these observations are linked to the OT system is an open question.

4. Channel-mediated OTR effects (e.g., depolarizing effects shown in hippocampal neurons via OTR-mediated potassium channel closure (Tirko et al., 2018); Fig. 7, bottom left) may facilitate short-term (Marlin et al., 2015) and long-term (Ninan, 2011, Lin et al., 2012, Hu et al., 2021, Gur et al., 2014) plasticity, thereby mediating pup cue-directed learning i.e., the acquisition of appropriate maternal care). For a comprehensive review, please refer to Froemke and Young (2021).

**Fig. 8:**
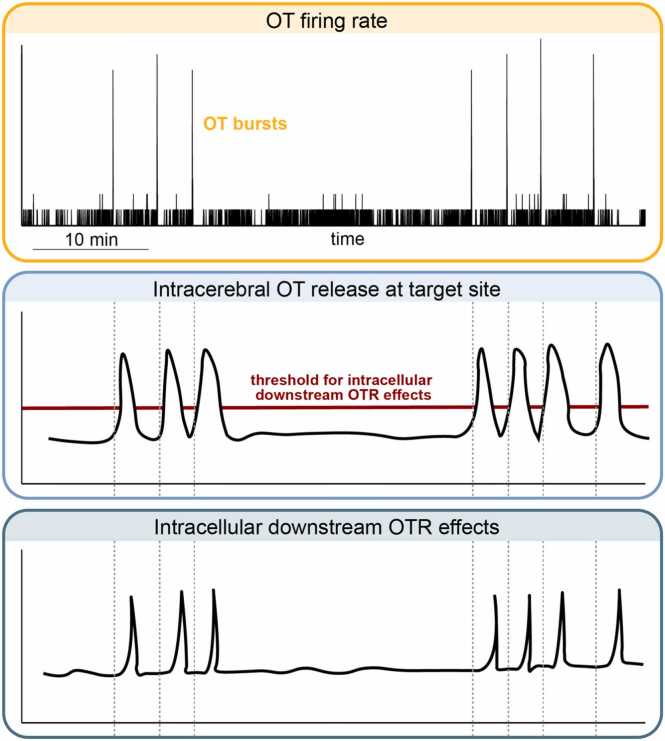
Non-linear OT release and switch-like OTR intracellular pathway responses following OT bursting. This arrangement of the OT system has not been directly demonstrated in a single experiment but is instead proposed as a hypothesis derived from the literature summarized above. Top trace: Real data showing the firing rate of a putative OT neuron from a lactating rat, provided by the authors. Middle and bottom traces: Hypothesized responses were drawn by the authors and are derived from the research summarized above.

In summary, OT exerts its effects across diverse brain regions, over multiple temporal scales and via distinct mechanisms. Similar to OT release, OTR signaling leads to non-linear responses, such as in the case of the MAPK pathway, which activates downstream effectors in a switch-like manner once a certain threshold has been crossed (Fig. 8). This suggests that certain OTR signaling pathways may only be enabled following OT bursting. Moreover, OT signaling may converge with other endocrine pathways, thereby boosting the non-linear responses described above. Milk ejection-inducing OT bursting may represent an important component of these cooperative effects and may therefore be a key factor in maternal health and disease. Future experiments combining investigations of multiple neuroendocrine pathways with dose-response analyses of OT concentrations and downstream effects will further refine our knowledge of how these complex OT functions mediate appropriate mother-child interactions.

## 3 Oxytocin, breastfeeding, and postpartum depression: interactions and clinical insights

### 3.1 Oxytocin levels and risk of postpartum depression

OT has been recognized as a potential modulator of maternal mood, stress regulation, and emotional bonding (Pedersen et al., 1982, Neumann, 2002, Windle et al., 2004, Strathearn et al., 2009, Grinevich et al., 2016, Neumann and Slattery, 2016). Disruptions of the OT system have been implicated in the pathophysiology of postpartum depression (PPD) (Skrundz et al., 2011), one of the most prevalent and clinically significant psychiatric disorders of the peripartum period that affects around 17% of women worldwide (Wang et al., 2021). PPD is associated with impaired mother-infant bonding, increased exposure to child maltreatment and other adverse childhood experiences (ACEs), and the intergenerational transmission of emotional and behavioral vulnerability (Choi et al., 2019, Lehnig et al., 2019, Suarez et al., 2023). In addition to its developmental consequences, PPD imposes a substantial economic burden, with affected households exhibiting markedly higher healthcare utilization and costs in the postpartum period (Epperson et al., 2020, Moore Simas et al., 2020).

To understand how alterations in the OT system may contribute to maternal mood disorders, it is first necessary to consider the broader etiological landscape of PPD. The disorder emerges from a complex interplay of hormonal, neuroendocrine, immune, genetic, and psychosocial influences that collectively determine stress responsiveness in the postpartum period (Bloch et al., 2000, Glynn et al., 2013, Bränn et al., 2017, Bauer et al., 2025). Against this etiological backdrop, a growing body of human research has examined whether variation in OT levels is associated with vulnerability to PPD symptoms.

Multiple human studies indicate that low peripheral OT levels are associated with an increased risk of PPD. A 2020 systematic review (16 studies) found that in 8 of 12 studies on endogenous OT measurements, women with lower plasma OT levels had more depressive symptoms (Thul et al., 2020). Similarly, longitudinal data suggest that diminished OT levels during pregnancy predict subsequent PPD, which implies a protective effect of robust OT levels. For example, in a prospective cohort study (Skrundz et al., 2011), found that lower OT concentrations in the third trimester were associated with higher Edinburgh Postnatal Depression Scale EPDS) scores postpartum. Complementing this, Jobst et al. (2016) reported that reduced OT levels during late pregnancy prospectively predicted depressive symptoms following childbirth. Extending these observations beyond plasma measures, converging evidence from salivary OT assessments further supports an inverse association between endogenous OT levels and early postpartum affective symptoms. In primiparous mothers, Shishido et al. (2019) reported a negative correlation between salivary OT concentrations and transient “maternity blues” scores, suggesting that higher OT availability may buffer against early mood disturbances. Similarly, Cevik and Alan (2021) found that salivary OT levels measured between the 30th and 38th gestational weeks demonstrated moderate predictive value for subsequent PPD symptoms. Together, these findings across biological compartments and peripartum time points are consistent with OT’s established role in stress attenuation and positive affect regulation.

**Fig. 9:**
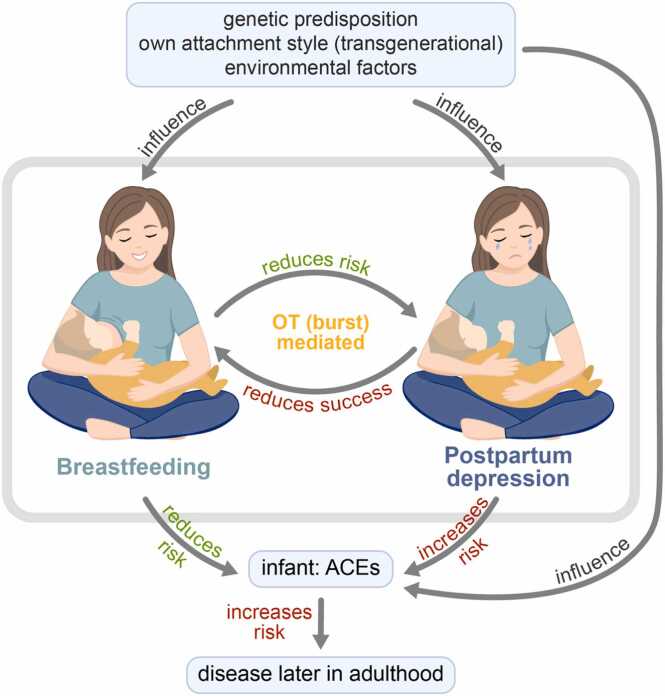
Influences of oxytocin on health and disease. Breastfeeding and postpartum depression (PPD) are interrelated and have complex origins (top) and consequences (bottom). PPD and breastfeeding are inversely related to each other. Women who successfully breastfeed are less likely to develop PPD symptoms, and conversely, women with PPD symptoms are less likely to successfully breastfeed. Note that while this bidirectional relationship is well established, the underlying mechanisms remain poorly understood. Children of mothers with PPD more frequently experience neglect and other adverse childhood experiences (ACEs), which in turn are associated with an increased propensity to develop mental and physical disorders later in life. The likelihood of developing PPD symptoms depends on genetic predisposition, individual attachment style, and environmental factors. Note that arrows indicate general trends cited in the main text but do not reflect the strength of evidence or imply strict causal relationships. The direction of influence can be partially inferred from intervention studies. This review focuses on the interplay between breastfeeding, OT bursting, and postpartum depression (gray rectangle). It does not cover influencing factors, such as genetic predisposition or attachment styles (top), nor PPD-associated outcomes, such as ACEs (Hughes et al., 2017, Felitti et al., 1998) and their consequences (e.g., disease later in adulthood, bottom).

More recent work has further strengthened this association by leveraging multi-compartment and prospective study designs that more directly index central OT signaling. In a longitudinal cohort of women undergoing elective cesarean delivery, Chen et al. (2022) simultaneously measured OT concentrations in the cerebrospinal fluid (CSF), plasma, and saliva, and found that levels in all three compartments were significantly lower in women who subsequently developed PPD. Notably, CSF OT demonstrated the strongest predictive performance (AUC ≈ 0.89), which supports the notion that CSF OT availability may be particularly relevant for postpartum mood outcomes. Given evidence that neuropeptides can diffuse bidirectionally between the CSF and brain parenchyma (Dogterom et al., 1977, Charles, 2001, Iliff et al., 2012), the authors argued that CSF OT may more faithfully reflect central release dynamics than peripheral measures alone. Importantly, salivary OT correlated more closely with CSF than plasma levels, which suggests that saliva may provide a practical peripheral proxy for central OT activity when CSF sampling is not feasible. Consistent with this interpretation, Lu et al. (2025) reported that lower salivary OT levels measured within the first two days postpartum independently predicted PPD at four weeks, even after accounting for early EPDS scores and perceived social support (AUC ≈ 0.82). Together, these findings indicate that reduced OT availability, particularly within central or centrally coupled compartments during critical peripartum windows, may confer heightened vulnerability to PPD outcomes (Fig. 9).

### 3.2 Breastfeeding as a driver of oxytocin bursting and maternal mood regulation

Clinically, breastfeeding initiation, continuity, and exclusivity are consistently associated with more favorable postpartum mood outcomes across diverse populations. Large prospective cohorts indicate that early initiation of breastfeeding, typically within the first hours after birth, is linked to a substantially lower risk of PPD symptoms, with estimates suggesting roughly a 40–50% reduction in PPD likelihood compared to delayed or unsuccessful initiation (Shen et al., 2022, Roy and Chaillet, 2024). Conversely, discontinuation of breastfeeding within the first postpartum week is associated with markedly increased PPD risk, including an approximate twofold elevation in depressive symptoms in some cohorts, particularly among older mothers and those delivering by cesarean section (Chiu et al., 2020). Beyond initiation, breastfeeding continuity and exclusivity appear to further shape risk trajectories: continued breastfeeding for at least three months is associated with lower odds of moderate-to-severe PPD across cultural contexts (Figueiredo et al., 2021, Al Rajabi et al., 2025). Importantly, longitudinal evidence suggests that exclusive breastfeeding may be especially protective among women with elevated vulnerability, such as those with prenatal depressive symptoms, where sustained breastfeeding is associated with attenuation of postpartum symptom severity (Figueiredo et al., 2021). Although these associations likely reflect bidirectional influences between maternal mood and feeding behavior, their consistency across large cohorts and settings supports the view that sustained engagement in breastfeeding-related behaviors may reinforce neuroendocrine processes that buffer stress and support mood regulation during the postpartum period (Fig. 9).

However, breastfeeding-related mood benefits are not uniform and can depend strongly on the emotional and contextual quality of the breastfeeding experience. Across feeding styles, several studies suggest that depressive symptoms are more closely linked to maternal stress, perceived control, and satisfaction with feeding than to feeding method alone. Mixed feeding and early breastfeeding difficulties are consistently associated with greater emotional tension, concerns about infant satiety, and impaired bonding, despite similar overall depression scores across groups (Kossakowska and Bielawska-Batorowicz, 2022, Carvalho Hilje et al., 2024). Qualitative evidence further indicates that pain, logistical challenges, and unmet expectations can attenuate the calming effects typically associated with breastfeeding (Stelson et al., 2021). Notably, longitudinal population data show that breastfeeding effects on maternal mood are strongly moderated by prenatal intention, with the lowest PPD risk observed when planned breastfeeding is achieved and the highest risk observed when women intend to breastfeed but are unable to do so (Borra et al., 2015). Together, these findings suggest that breastfeeding supports maternal mood most effectively when suckling-evoked OT bursts occur within a predictable and emotionally congruent context. When feeding is stressful or conflicted, OT-mediated stress buffering may be diminished.

An important question regarding the role of OT bursts in stress buffering is whether temporally locked, suckling-induced OT responses better predict PPD symptoms than basal OT levels, i.e., OT samples obtained outside breastfeeding events. We identified three studies from the same group that investigated the relationship between time-resolved OT levels during breastfeeding and PPD, using similar but not identical methodologies (Table 1).

**Table 1:** Characteristics of studies investigating time-resolved OT responses during breastfeeding and their interactions with PPD.

**Study**	**No. of participants (N) for the 2-month postpartum period**	**PPD** **assessment** **tool**	**Sample** **specimen**	**Sampling frequency**
Stuebe et al. (2013)	39	EPDS	Plasma OT	Baseline and 1, 4, 7 and 10 min after feeding onset
Cox et al. (2015)	39	EPDS	Plasma OT	Baseline and 1, 4, 7 and 10 min after feeding onset
Whitley et al. (2020)	145	Beck Depression Inventory	Plasma OT and saliva OT	Baseline and 1, 4, 7 and 10 min after feeding onset

As shown in Table 1, studies vary in their methodologies, making it difficult to compare time-resolved OT measurement studies with those that did not sample during breastfeeding. Both Stuebe et al. (2013) and Cox et al. (2015) reported that mothers with depressive symptoms had lower plasma OT levels. Similarly, the study mentioned earlier (Chen et al., 2022) reported lower salivary, plasma, and CSF OT levels in symptomatic mothers. However, they did not sample during breastfeeding. The closest match between studies investigating either OT during breastfeeding or OT outside breastfeeding appeared to be Stuebe et al. and Chen et al. (2022), as both reported correlation values between OT levels and PPD symptomatology. In Stuebe et al., OT levels were most strongly correlated with PPD symptoms 7 min after suckling onset (r = -0.5), whereas the correlation was weaker and not significant at baseline (r = -0.22). The latter correlation value is consistent with results from Chen et al., who reported a similarly weak but significant interaction in a non-breastfeeding setting (r = -0.24). These comparisons should be interpreted with caution, particularly because the initial findings of Stuebe and Cox et al. were not replicated in a high-powered follow-up study conducted by the same group (Whitley et al., 2020). To our knowledge, the latter study is the largest and most recent investigation of the interaction between time-resolved OT responses during breastfeeding and maternal depressive symptoms. Although methodological differences exist compared to earlier studies, the negative findings of Whitley et al., 2020 suggest that the relationship between suckling-induced OT responses and PPD is not straightforward. As if to fuel this controversy, a recent study reported that subclinical EPDS scores were negatively correlated with OT plasma level responses in breastfeeding women (Minami et al., 2025). Another study measuring OT responses during breastfeeding similarly reported lower OT responses in anxious mothers (Nagahashi-Araki et al., 2022). These two recent studies thereby support the initial findings by Stuebe and Cox et al. The contradicting literature on this topic likely reflects methodological variability, but the specific confounding factors remain difficult to identify. We speculate that some of these confounding factors remain unidentified, partly because the relationship between peripheral OT levels and central release in specific target areas is not yet fully understood. Animal studies may provide hints toward this issue. In a seminal study, Zhang et al. (2021) showed that pituitary-projecting magnocellular OT neurons also project to forebrain areas and can modulate behavior. This suggests that OT plasma levels and centrally released OT are linked. However, as shown in Hasan et al. (2019), different subpopulations of OT neurons can be activated in different contexts, suggesting that central and peripheral release could be decoupled. Animal studies investigating the fundamental relationship between OT bursting and central OT release may shed light on its connection to PPD. While many questions on this topic remain unresolved, the following section outlines some of the known cellular and molecular pathways that may help explain the complex interaction between OT, breastfeeding, and PPD.

## 4 Possible OT-dependent pathophysiological mechanisms underlying PPD

As summarized in Section 2, physiological recordings of OT neurons revealed that milk ejections are preceded by unique OT bursts that may lead not only to substantial OT secretion into the bloodstream but also to release in various brain areas. The reviewed literature suggests that burst-driven increases in OT secretion induced by infant suckling, as opposed to phasic release, may play an important role in maternal mental health during the postpartum period. However, the exact contributions of burst-associated OT release across different brain areas to maternal mental disorders remain unclear. In this section, we first review what is known about the etiology of PPD, the most prevalent postpartum disorder, and subsequently speculate on how OT bursting and PPD may interact bidirectionally at a mechanistic level.

### 4.1 OT-dependent circuits, stress and PPD

Elevated OT levels due to infant suckling-induced OT bursts likely affect a variety of brain areas, each with specialized functions in maternal behavior (Fig. 1, Fig. 2). Some of these areas form emotion-related circuits that regulate state changes and can serve as potent switches, turning maternal care on or off (Kohl and Dulac, 2018, Láng et al., 2024). Perturbation of these circuits leads to reduced maternal care, which is a hallmark of PPD. However, the absence of maternal care is not a clear-cut indicator of PPD: reduced maternal care is also evident in mothers who don’t suffer from PPD (Hitzler et al., 2022). Further, a recent study using mice (Witchey et al., 2024) showed that while genetic knockdown (KO) of OTRs in the nucleus accumbens reduced maternal care, the authors did not observe differences in behavioral readouts assessing depression-like phenotypes. Conversely, OTR-KO in the posterior paraventricular thalamus (PVT) did not impair maternal care, but these animals displayed depression-like behaviors. These observations support the idea that a lack of maternal care or motivation per se does not necessarily determine overall maternal mental health.

**Fig. 10:**
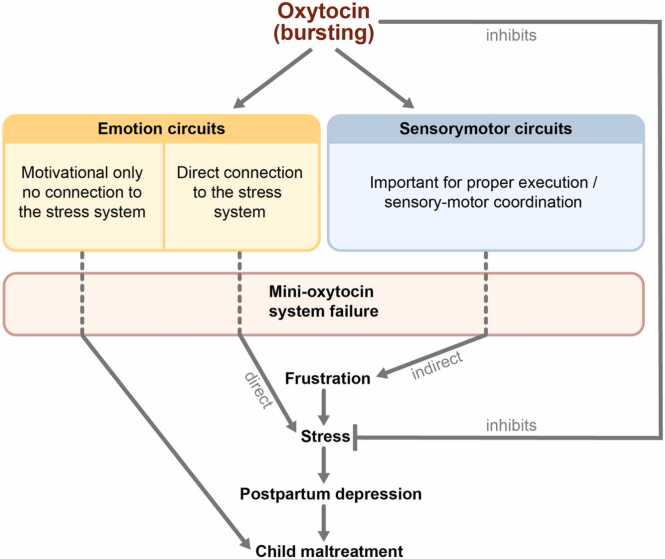
Proposed circuits potentially involved in the development of PPD. Left: Direct effects of Mini-OT system failures on maternal behavior via emotion-related circuits. Note that impairment of maternal motivation for caregiving alone may not be sufficient to drive PPD. However, if circuits are connected to the stress system, they can contribute to PPD. See Section 4.2 for a review of concrete example circuits. Right: Indirect effects of Mini-OT system failures on stress and PPD through sensorimotor deficits. Note that this relationship is understudied (mainly supported by indirect evidence; see Section 4.3) and therefore requires further experimental validation.

We speculate that the development of PPD is highly dependent on the affected neuronal circuits that control different aspects of maternity. In particular, we propose that circuits that *directly* or *indirectly* affect the stress axis are more likely to contribute to PPD symptoms than circuits not associated with the stress system, even if the latter are well-known for driving maternal behavior (Fig. 10, left). If mothers perceive their care negatively, they may experience cognitive stress and subsequent PPD symptoms. This may occur due to sensorimotor deficits that impair proper maternal caregiving while maternal motivation remains intact. This combination may be especially frustrating for mothers and could be an important mechanism for PPD development (Fig. 10, right).

In the following sections, we elaborate on *direct* and *indirect* pathways (Fig. 10) that bidirectionally link the OT and stress systems.

### 4.2 *Direct* pathways between the OT system and limbic areas

#### 4.2.1 OT → stress system → PPD attenuation

**Fig. 11:**
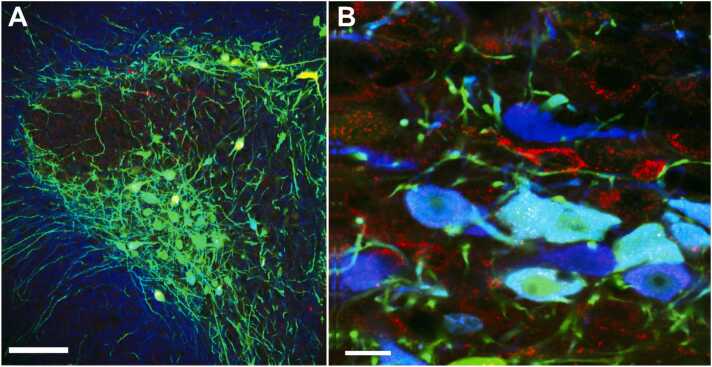
Distribution of OT- and CRH-expressing neurons and processes within the rat PVN. A. Overview of the rat PVN following infection with a cell type–specific rAAV carrying a 1.9 kb OT promoter driving the fluorescent marker Venus (green). Sections were stained with antibodies against GFP (green), CRH (red) and OT (blue). Scale bar = 200 μm. B. Higher-magnification view of the PVN showing a dense plexus of OT- and CRH-expressing neuronal cell bodies and their processes in close proximity. Scale bar = 20 μm. Valery Grinevich, unpublished data.

The OT and stress systems are highly intertwined (see reviews (Uvnas-Moberg et al., 2024, Onaka and Takayanagi, 2019, Zhang et al., 2023, Neumann, 2002, Winter and Jurek, 2019)). Corticotropin-releasing hormone (CRH)-expressing neurons are surrounded by OT cells in the PVN (Fig. 11).

**Fig. 12:**
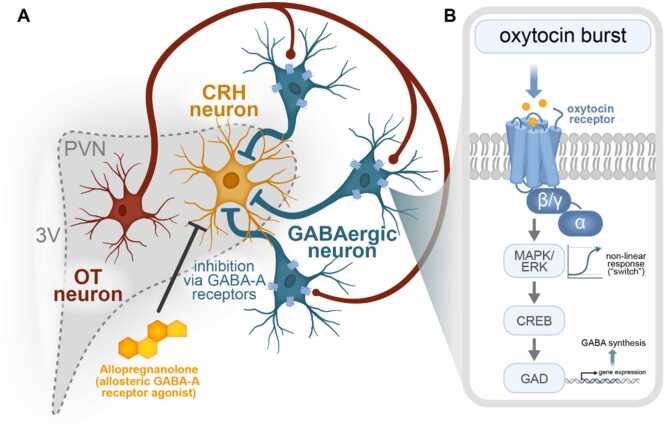
Proposed direct pathways linking OT bursting and stress underlying the protective effects of breastfeeding against PPD. A. The circuit is based on references cited in Section 4.2.1 and partially adapted from (Winter and Jurek, 2019, Takahashi, 2020) (with permission; for (Takahashi, 2020), no permission was required due to a CC BY license). We propose that allopregnanolone (ALLO) may target the same CRH neuron population that is also indirectly inhibited by OT. Note that, to our knowledge, the exact identity and location of CRH-inhibiting GABAergic neurons have not yet been determined. B. Hypothetical molecular mechanism of OT-driven increases in inhibitory signaling. GABAergic interneurons may increase GABA synthesis via CREB-dependent upregulation of GAD expression (Sanchez-Huertas and Rico, 2011), driven by OTR-dependent MAPK/ERK activation (Devost et al., 2022, Xing et al., 1996).

However, despite the close apposition of their somata and processes, direct communication

between these two cell types has not yet been demonstrated. In contrast, OT has been reported to inhibit the HPA axis (Neumann et al., 2000) indirectly via GABAergic signaling in the PVN (Windle et al., 2004, Takahashi, 2020). One idea put forward by Takahashi 2021 (Takahashi, 2020) as well as by Winter and Jurek (2019) is that OT exerts its effects on GABA-A receptor-expressing CRH neurons via connectivity with PVN-surrounding GABAergic neurons (Boudaba et al., 1996), which in turn project back to the PVN and inhibit GABA-A receptor-expressing CRH neurons (Takahashi, 2020) (Fig. 12A; for a comprehensive review on GABAergic control of the HPA axis, see (Camille Melón and Maguire, 2016)). While this circuit requires further experimental validation (e.g., identifying the precise identity of the GABAergic population using modern circuit mapping approaches), it exemplifies how an OT-dependent circuit may regulate the HPA axis. This is supported by observations showing that OT can reduce anxiety-like behaviors via GABA-A receptor activation and can dampen corticosterone levels as well as CRH neuron activity (Smith et al., 2016). Furthermore, CRH expression is inhibited by OT via GABA-A receptors (Bulbul et al., 2011).

In line with these observations, OT action affects maternal stress-related emotional regulation. A study by Bosch et al. (2005) showed that OT mediates maternal aggression, which is associated with anxiety. More anxious rat mothers were more aggressive, especially when OT was present in the hypothalamus or the amygdala (Bosch et al., 2005). Similarly, OT prevents maternal freezing following predatory cues (Febo et al., 2009, Rickenbacher et al., 2017), an adaptive behavioral mechanism to ensure offspring survival.

While these studies suggest that OT inhibits the stress system, potentially via CRH inhibition, experiments directly probing interactions between the OT and CRH systems have primarily been conducted in non-lactating animals. The only study we found in this direction investigated OT antagonist (OTA)-mediated CRH responses in lactating animals (Neumann et al., 1999). In this study, OTA infusion increased CRH levels, but only in virgin and non-lactating animals. Further experiments are required to clarify the exact brain mechanisms by which OT exerts its effect on the stress system during lactation.

It will also be interesting to investigate molecular changes of CRH neurons and upstream GABAergic populations during transition into motherhood. Suckling-induced OT bursts may have gene expression effects via intracellular pathways as described in Section 2. As reviewed above, sufficient OTR activation (potentially facilitated by OT burst-driven non-linear OT release) can activate the MAPK/ERK signaling pathway. ERK activates the transcription factor CREB (Xing et al., 1996), which in turn has been shown to increase glutamate decarboxylase (GAD) expression (Sanchez-Huertas and Rico, 2011), the rate-limiting enzyme catalyzing GABA synthesis. Therefore, OT may increase inhibitory tone on CRH neurons by driving OTR signaling-dependent increases in GABA synthesis (Fig. 12B). Such OT-induced increases in GABAergic control due to suckling-induced OT bursting may not be limited to the hypothalamus but may also exist in other stress-related effectors, such as amygdala output neurons controlled by OT-dependent local inhibition (Knobloch et al., 2012).

GABAergic control of the stress system has also been investigated from a different angle. Neuroactive steroids, such as allopregnanolone (ALLO), are positive allosteric modulators of GABA-A receptors (Abramian et al., 2014, Laverty et al., 2017). A recent systematic review shows that ALLO levels drop sharply after parturition (Grötsch and Ehlert, 2024). This decrease is thought to constitute one contributing pathophysiological factor for PPD by reducing the GABAergic “brake” on the HPA system (see (Meltzer-Brody and Kanes, 2020) for review). We speculate that such ALLO-driven GABAergic brake is not restricted to the hypothalamus but may act in other brain regions. Indeed, ALLO has been subject to various clinical trials with promising outcomes for PPD symptoms (reviewed in (Patterson et al., 2024)).

We speculate that the antidepressant(-like) actions of OT and ALLO may be mechanistically explained by their shaired effects on CRH neurons, but to our knowledge, this has not yet been investigated. If both indeed converge on the HPA system (as shown in Fig. 12A), synergistic effects of synthetic OT or breastfeeding interventions with ALLO may be possible. However, as ALLO has been shown to modulate OT expression itself (Blyth et al., 2000), further preclinical research is required to elucidate precise OT-ALLO interactions.

#### 4.2.2 PPD → stress system → OT attenuation

**Fig. 13:**
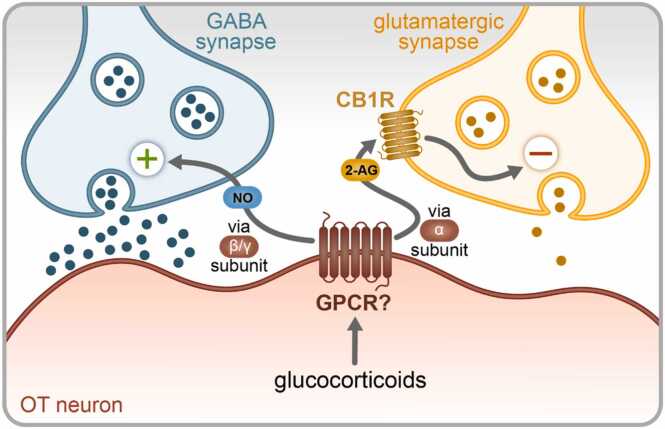
Mechanisms that may lead to reduced OT bursting due to stress. Glucocorticoids likely activate postsynaptic GPCRs, which retrogradely increase GABAergic presynaptic activity and inhibit glutamatergic presynaptic activity via nitric oxide (NO), or 2-arachidonoylglycerol (2-AG)-mediated cannabinoid receptor 1 (CB1R) signaling (Di et al., 2005, Di et al., 2009). Both pathways lead to net inhibition of OT neurons. Partially adapted from (Joëls et al., 2024) (with permission).

One important observation is that the OT rise following infant suckling (“OT response”) is blunted in depressed mothers, whereas corticosterone levels are elevated (Stuebe et al., 2013, Cox et al., 2015). In healthy, but not depressed, mothers, OT and corticosterone levels are negatively correlated (Cox et al., 2015). This suggests that in mothers with PPD, the OT circuits described in the previous section do not sufficiently regulate the HPA axis. Potential mechanisms include reduced OT secretion (Vilela and Giusti-Paiva, 2011) (and potentially reduced bursting) and reduced GABA-mediated firing of magnocellular PVN neurons (Di et al., 2005), both of which are driven by glucocorticoids. This appears to represent a vicious cycle: PPD → increased stress hormones → reduced OT secretion and firing → diminished HPA axis control → further increases in stress hormones. This suggests that breastfeeding impairments associated with elevated stress hormone levels may result from glucocorticoid-driven impairment of OT burst firing. This idea is supported by observations showing both rapidly reduced excitation and increased inhibition of PVN magnocellular neurons by glucocorticoids (Di et al., 2005, Di et al., 2009) (Fig. 13).

The exact contribution of PPD-associated HPA axis elevation to OT burst firing, as well as the cellular nodes of the circuits discussed above, remains to be clarified. We would like to particularly emphasize the knowledge gap regarding the identity of GABAergic neurons controlling CRH and OT neurons, respectively.

### 4.3 Indirect pathways: sensory-motor areas and stress

#### 4.3.1 OT → sensorimotor circuits → Stress/PPD

As outlined in Section 3, many studies report that early tactile sensory contact is important for successful breastfeeding. However, the exact underlying mechanisms remain largely unexplored, and both theoretical frameworks and empirical data on whether and how OT affects sensorimotor coordination during breastfeeding or nursing are scarce. One study showed that mothers with sensory over-responsiveness discontinued breastfeeding earlier than control mothers (Freund-Azaria et al., 2024), although this over-responsiveness was more strongly linked to increased pain than to sensorimotor deficits. Nevertheless, sensorimotor coordination (e.g., holding the infant correctly and responding appropriately to sensory cues) is critical for successful breastfeeding (Degefa et al., 2019, Yilak et al., 2020, Parashar et al., 2015, Vinther et al., 1997, Dongre et al., 2010, Agarwal et al., 2009, Davra et al., 2022, Tiruye et al., 2018, Safayi et al., 2021, Alemie et al., 2023). As sensorimotor impairment can lead to stress (reviewed in (Erskine et al., 2024)), and breastfeeding can be protective against PPD (see Section 3), sensory deficits may contribute to PPD development.

Recent findings demonstrate OT’s role in shaping sensorimotor function. OT in the auditory cortex is necessary for appropriate maternal care (Carcea et al., 2021, Marlin et al., 2015, Schiavo et al., 2020). These studies use pup retrieval as a behavioral readout rather than nursing. Impaired retrieval may lead to stress if mothers are aware of their deficits; however, this will need to be demonstrated in future studies.

Furthermore, OT improves olfactory processing by increasing the inhibition-driven signal-to-noise ratio of sensory inputs in the olfactory cortex, leading to enhanced conspecific recognition (Oettl et al., 2016). Similarly, OT increases inhibitory drive in the visual cortex, while its role in modulating inhibition in the somatosensory cortex remains unclear (Maldonado et al., 2021). Although these studies did not investigate OT and sensory processing during maternity, it is tempting to speculate that OT may tune all sensory cortices toward optimizing maternal behavior. In this context, two studies are particularly noteworthy. Xerri et al. and Rosselet et al. demonstrated receptive field sharpening in the ventral trunk area of the somatosensory cortex (Rosselet et al., 2006, Xerri et al., 1994). As suggested by Valtcheva and Froemke, 2019, these neuroplastic mechanisms may be driven by OT (Valtcheva and Froemke, 2019), therefore improving tactile acuity and nursing.

In the motor domain, one study demonstrated effects of OT on motor neurons in the spinal cord (Pearson et al., 2003); however, to our knowledge, studies on OT-dependent circuits in the motor cortex, striatum, or cerebellum remain limited or inconclusive (Shen et al., 2023).

Finally, stress impairs perceptual learning (Dinse et al., 2017) and leads to reduced cortical plasticity (Gellner et al., 2022, Daw et al., 1991, Sale et al., 2008) as well as motor learning disabilities (Gellner et al., 2022) (see also (Anderson et al., 2019) and (Martins et al., 2024) for comprehensive reviews). In turn, as outlined in the section above, sensorimotor impairments can lead to maternal stress and PPD. Thus, the relationship between sensorimotor function and stress is bidirectional.

In summary, studies show that OT modulates sensorimotor circuits across various modalities, thereby enhancing the processing of social cues. In mothers, this may dampen stress during the postpartum period, which is characterized by both physical and psychological challenges across emotional and sensorimotor domains. To our knowledge, however, empirical data are lacking, and future studies may uncover the contributions of different sensorimotor areas to stress regulation.

## 5 Conclusion and outlook

Milk ejection is preceded by bursts of action potentials in OT neurons, which result in massive, momentary release of OT into the bloodstream, and most likely also into the brain. The latter is often overlooked when discussing mechanisms concerning protective effects of OT. Despite this well-documented physiological phenomenon, research on the mechanisms underlying OT bursts, as well as the effects of breastfeeding-induced OT bursts and their concurrent release across brain areas, has received limited attention. Women may differ in their degree of OT and OTR expression levels, as well as in the degree or frequency of OT burst generation. These factors may positively influence breastfeeding success by directly inhibiting the stress system or indirectly preventing stress through sensorimotor circuit optimization. Dysregulation of these processes may contribute to the development of clinical conditions, such as PPD and ACEs.

**Fig. 14:**
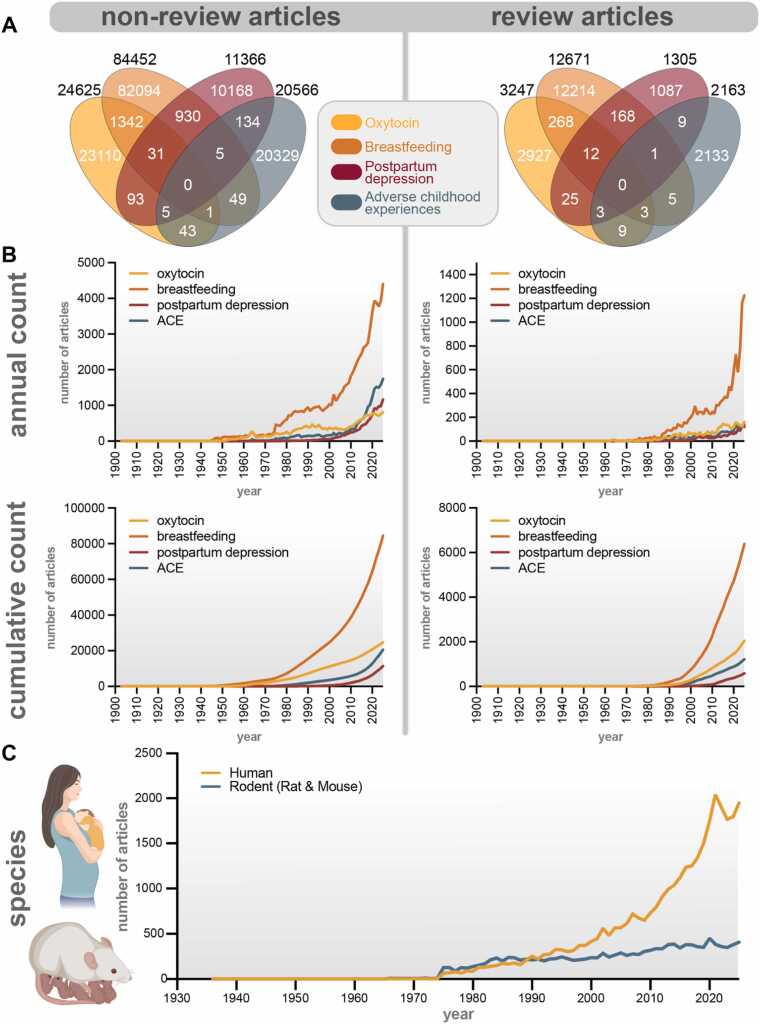
Publication trends by topic and publication type. A. Left: The Venn diagram shows the number of non-review articles associated with each topic and their intersections. Right: Same as the left, but for review articles. Note that although studies focusing on single topics are abundant, studies addressing intersecting topics are much rarer. B. Top left: Annual counts of non-review articles for each topic. Bottom left: Cumulative counts of non-review articles for each topic. Top right: Same as the top left, but for review articles. Bottom right: Same as the bottom right, but for review articles. C. Contrast between human and rodent studies on breastfeeding. Note that the surge in human studies on breastfeeding is not reflected in the rodent literature.

Future human experiments on breastfeeding could build on mechanistic findings from animal research to evaluate potential translational applications. Based on our PubMed literature search, we want to point out that while OT, breastfeeding, PPD, and ACEs have each been extensively studied in isolation, there is a shortage of more “holistic” studies integrating these topics (Fig. 14A). Although such studies would be desirable to be conducted in humans, there are significant practical hurdles, such as subject recruitment for longitudinal and transgenerational studies. Furthermore, OT measurement and administration, as well as neuronal circuit investigations, remain limited in humans. We therefore emphasize that animal studies are required to complement future human experiments. There appears to be a surge in human studies on breastfeeding (Fig. 14B; see Section 6 for search details), based on keywords such as breastfeeding, lactation, suckling, nursing, and milk ejection. This surge may reflect growing recognition within the clinical research community of the importance of breastfeeding neurobiology for mental health (although other factors have likely also contributed; see (Victora et al., 2016, Rollins et al., 2016)). This trend has not yet been reflected to the same extent in the animal research community (Fig. 14C). We hope that this will change through closer interactions between clinical and basic scientists. We suppose that a better fundamental understanding of the neural substrates underlying OT bursting and respective downstream effects will be critical for future translational trajectories in the field of maternal mental health.

As a final note, we emphasize that this review does not address the upstream mechanisms underlying OT bursting. Elucidating these processes will be equally important for advancing our understanding of postpartum maternal physiology and related disorders.

## 6 Publication trends analysis method

### 6.1 Data acquisition

The PubMed database was queried via the NCBI E-utilities API. PMIDs were retrieved through the ESearch endpoint, with queries restricted to the title/abstract field ([tiab]) and limited to publication years from 1900 through 2025. The search was conducted in February 2026 to ensure comprehensive coverage of publications indexed for the year 2025. Four topic-specific query strings were employed:

Oxytocin: oxytocin[tiab]

Breastfeeding: (breastfeeding[tiab]) OR (breast feeding[tiab]) OR(breastfed[tiab]) OR (breast-feeding[tiab]) OR(lactation[tiab]) OR (lactating[tiab]) NOT (cow[tiab]) NOT (cows[tiab])

Postpartum depression: (postpartum depression[tiab])OR(post-partum depression[tiab])OR(postnatal depression[tiab])OR(post-natal depression[tiab])OR(perinatal depression[tiab])OR(peri-natal depression[tiab])OR(peripartum depression[tiab])OR(peri-partum depression[tiab])OR(postpartum affective disorder[tiab])OR(post-partum affective disorder[tiab])OR(postnatal affective disorder[tiab])OR(post-natal affective disorder[tiab])OR(peripartum affective disorder[tiab])OR(peri-partum affective disorder[tiab])OR(perinatal affective disorder[tiab])OR(peri-natal affective disorder[tiab])OR(postpartum mood disorder[tiab])OR(post-partum mood disorder[tiab])OR(postnatal mood disorder[tiab])OR(post-natal mood disorder[tiab])OR(peripartum mood disorder[tiab])OR(peri-partum mood disorder[tiab])OR(perinatal mood disorder[tiab])OR(peri-natal mood disorder[tiab])

Adverse childhood experiences: (adverse childhood experiences[tiab])OR(child abuse[tiab])OR(child maltreatment[tiab])OR(child neglect[tiab])

Publication types and abstracts were then acquired via the ESummary and EFetch endpoints, respectively. Records bearing any of the following publication types were excluded:

'News', 'Newspaper Article', 'Biography', 'Comment', ‘'Consensus Development Conference', 'Editorial', 'Guideline', 'Interview', 'Legislation', 'Letter', 'Overall', 'Patient Education Handout', 'Practice Guideline', 'Published Erratum', and 'Retracted Publication'.

Articles without publication types were included, as were articles without abstracts. Articles with ‘Review’ publication type were treated as review articles.

In total, 157,198 unique PMIDs met the inclusion criteria and were considered valid articles. Of these, 18,864 were classified as review articles and 138,334 as non-review articles. These counts refer to unique PMIDs across all queries. For topic-specific analyses, articles could be counted in multiple categories if they were retrieved by more than one query term.

### 6.2 Data analysis

Articles were classified as either review or non-review based on the presence of the publication type “Review” in PubMed metadata. All other eligible records were categorized as non-review articles. For each of the four topic-specific queries, the total number of retrieved articles was quantified separately for review and non-review articles. Overlaps between topics were determined by intersecting PMIDs across query results, and these intersections were visualized using Venn diagrams. Annual publication counts were calculated based on publication year metadata for each topic and article type. Cumulative publication counts were computed by summing annual counts across years from 1900 to 2025.

Species-specific analysis was restricted to records with available abstracts. Of 160,395 valid articles, 19,243 records lacking abstracts were excluded from this analysis. Among articles with abstracts, keyword-based classification was performed. Articles were categorized as human-relevant if the abstract contained the terms “human,” “woman,” or “women.” Articles were categorized as rodent-relevant if the abstract contained the terms “rat,” “rats,” “mouse,” or “mice.” Articles could be counted in both categories if both sets of terms were present.

## Declaration of Generative AI and AI-assisted technologies in the writing process

During the preparation of this work, the authors used ChatGPT, in addition to conventional tools such as PubMed and Google Scholar, to assist with literature searches. All outputs were reviewed and edited by the authors as needed, and the authors take full responsibility for the content of the published article.
